# The Book World of Medicine and Science

**Published:** 1896-11-28

**Authors:** 


### The Book World of Medicine and Science.

The Increase of General Paralysis in England and Wales: Its Causation and Significance. By R. S. Stewart, M.D., D.P.H., Deputy Medical Superintendent Glamorgan County Asylum, Bridgend. (South Counties Press, Lewes.)

In this interesting pamphlet Dr. Stewart directs our attention to the great part played by alcoholic and other excesses in the production of progressive paresis. Dr. Stewart forcibly points out that the increase of this peculiarly fatal disease is to be traced chiefly to causes which are to a very large extent controllable, and that the only direction in which a remedy is to be sought is in that of prevention. The Hospital has persistently dinned into the public ear that until preventive measures be enforced by an enlightened public opinion it is vain to hope for any elimination of insanity or of the burden which its existence imposes upon the community. Not only do the insane crowd our institutions, but there remains unsequestered a large class of degenerates, the products of neurotic families, dangerous to society, by reason of their ability to entail by heredity, intensified deficiencies in their progeny. A closer acquaintance with physiological and psychological laws will tend in the direction of prevention, familiarising the student with the normal activities of the physical and mental forces and teaching how best to develop and preserve them; this will ensure more perfect beings, and will invite a progeny more free from ancestral taint. Reference to the reports of the Lunacy Commissioners shows how great a part in the production of insanity is played by these two factors—heredity and alcoholic excess. Asylum physicians, by the very nature of their position as State servants, are peculiarly fitted to impress upon the public mind these stern truths. We welcome Dr. Stewart's contribution to the literature of the subject.

Traitement des Suppurations Pelviennes (Treatment of Pelvic Suppurations). By Eugène Doyen. (Paris: Institut International de Bibliographie Scientifique. 1896. Pp. 78, with 19 illustrations.)

This brochure contains four papers which Doyen (of Reims) read at the International Congress of Gynæcology and Obstetrics held at Geneva in September; they have been reprinted from the Archives Provinciales de Chirurgie. The first paper describes Doyen's method of vaginal hysterectomy for suppurative and inflammatory conditions of the uterine appendages. He claims that for the majority of these cases the vaginal route is better than the abdominal one, for laparotomy leaves behind the uterus, which is the original source of the infection. He says that his method of removing the uterus and appendages possesses many advantages over that advocated by Pèan, and in so far as the opening into Douglas's pouch (posterior colpotomy) permits of an examination of the condition of the ovaries and tubes previous to their removal, this is true. We think, however, that he underrates the risks of rupturing the intestines in separating adhesions when operating per vaginam. He also advocates posterior colpotomy for the evacuation of purulent pelvic foci—a method that deserves more attention in England than it has hitherto received. This article also contains a vindication of Doyen's claim to the priority of having first performed this operation for such conditions. The second paper describes a valuable modification of his operation of total abdominal hysterectomy. The death-rate in the hands of several operators has only been 4·7 per cent., which is as low as that of ovariotomy. No forceps are used to control the hæmorrhage, but the vessels in the broad ligament are temporarily seized by the fingers; the time of operation need not exceed twenty-five minutes. Both papers are well illustrated. There are two other short papers on "An Operation for Backward Displacements of the Uterus" and "The Best Way of Closing the Abdominal Wall after Laparotomy."

